# Iodine status, and knowledge about iodine deficiency disorders in adolescent school girls aged 14-19 years, 2016

**DOI:** 10.15171/hpp.2019.10

**Published:** 2019-01-23

**Authors:** Zahra Heidari, Seyed Rafie Arefhosseini, Mehdi Hedayati, Elnaz Vaghef-Mehrabany, Mehranghiz Ebrahimi-Mameghani

**Affiliations:** ^1^Student Research Center, Tabriz University of Medical Sciences, Tabriz, Iran; ^2^Nutrition Research Center, Tabriz University of Medical Sciences, Tabriz, Iran; ^3^Cellular and Molecular Research Center, Research Institute for Endocrine Sciences, Shahid Beheshti University of Medical Sciences, Tehran, Iran; ^4^Social Determinant of Health Research Center, Tabriz University of Medical Sciences, Tabriz, Iran

**Keywords:** Iodine deficiency, Iodized salt, Knowledge, Adolescents

## Abstract

**Background: ** Adequate iodine intake by women in child-bearing age affects fetus neurodevelopment during pregnancy. A majority of previous studies has investigated iodine status among children, and there is limited data on female adolescents who are more exposed to consequences of iodine deficiency (ID) in their near-future pregnancies; thus, we aimed to assess iodine status, and knowledge on iodine deficiency disorders (IDDs) among adolescent school girls (14-19 years old) in Shahriar, Iran.

** Methods: ** This cross-sectional study was conducted among 223 female students selected through multi-stage cluster sampling from 12 schools. Iodine and creatinine concentrations were measured in casual urine samples. Iodine content of household salts was also assessed.Data on intake of salt and iodine-rich food sources were collected applying a food frequency questionnaire (FFQ), and knowledge about iodine and IDDs were assessed by a questionnaire.

** Results:** Median and Mean (95% CI) concentrations of urinary iodine and creatinine were 129 µg/L, 137.62 µg/L (95% CI: 126.28, 148.95) and 1.72 g/L, 1.86 g/L (95% CI: 0.55-3.17),respectively. The frequency of mild, moderate and severe ID were 22.4%, 14.3% and 0%,respectively; 43.5% had adequate, and 3.1% had excessive urinary iodine levels. Mean saltiodine concentration was 21.69 (SD=10.56) ppm. Mean knowledge score was 12.7 (SD=3.44).About half of the students had a poor (25.1%) or fair (24.2%) knowledge about iodine deficiency.Adjusting for the confounders, no significant positive association was found between knowledge about iodine-rich food sources and goitrogens with urinary iodine excretion.

**Conclusion: ** Adolescent girls in Shahriar had relatively poor knowledge of iodine, and about one third of them suffered from ID.

## Introduction

Iodine is a trace element (micronutrient) which plays a vital role in synthesis of thyroid hormones.^1^ Two billion people worldwide including 285 million children have iodine deficiency (ID).^2^ Iodine comprises 65% of thyroxin (T4) and 85% of triiodothyronine (T3) hormones.^3^ Thyroid hormones are crucial for growth and development of all body tissues throughout life. They are particularly essential for brain and neural system development during embryonic period. Therefore, it is critical to have adequate intake of iodine during pregnancy, to prevent irreversible consequences of ID.^4^ A study in England in 2013 showed that children born to mothers with urine iodine level of 50-150 µg/L had low IQ and cognitive function.^5^ Indeed, there is some evidence indicating that mild ID is common during pregnancy, which may result in reduced production of thyroid hormones, and consequently impaired fetal neurodevelopment.^6-8^

According to the World Health Organization (WHO) criteria, primary school children are at great risk of ID, and interventional programs for prevention of iodine deficiency disorders (IDDs) is crucial among them.^9^ Studies have shown that iodine status of children is not necessarily indicative of their parents’ nutritional status.^10^ For example, Fernando et al reported that urinary iodine concentration of children was twice higher than their mothers’ (115 µg/L vs. 57 µg/L).^11^ A number of studies have assessed ID among adolescents, emphasizing this group as a target group for IDD prevention programs.^12-16^

Adequate iodine intake is important among women of child-bearing age,^9^ and some studies have shown that pregnant women who had adequate iodine intake prior to conception, showed better status of thyroid hormones during pregnancy. A study in Italy showed that mothers who regularly consumed iodized salt for two years before conception, had better thyroid hormones status, higher urinary iodine concentrations (115 µg/L vs. 63 µg/L), and less thyroid dysfunctions (6.4% vs. 36.8%), compared to those who started consuming iodized salt from the beginning of their pregnancy.^17^ Our literature Review showed that most of the studies about iodine status have been conducted among primary school children and pregnant women; studies on adolescent and young girls (who are likely to conceive in near future) are limited. Therefore, it seems crucial that iodine status of this group of girls be assessed.

Shahriar, located in Tehran province in Iran with 744 210 people in 2017,^18^ has usually been characterized by high prevalence of ID, as iodine content of water and soil is low in this region.^17^ Results of the first national survey on IDD in 1969 revealed that the prevalence of goiter in Shahriar was 29% and 51% in males and females, respectively. Second national survey in 1983 showed that median urinary iodine (MUI) was 85 µg/L in Shahriar.^19,20^ Since 1989, extensive efforts have been made to control IDD in this area, through addition of iodine to salts, and implementation of public nutrition education programs emphasizing the importance of consuming iodized salt as well as the correct methods of preserving it.^21^ Since 1989, iodized salt has been widely available in Shahriar,^20^ but only one survey was conducted among school aged children in this region since then (1995), which indicated that MUI of the children was 185 µg/L.^20^ The Fourth National Surveillance in 2008 reported that MUI excretion remarkably decreased in some regions, though. For example, MUI excretion reduced from 190 µg/L to 94.1 µg/L, and the proportion of school aged children with urinary iodine excretion <50 µg/L increased from 11.2% to 20.8%, in Tehran.^22^ Similar results were obtained among school aged children in Shahriar region as well.^20^ These reports could be considered alarming.

To the best of our knowledge, no information is available on IDD, and also knowledge on iodine element as well as IDD complications among female adolescents; therefore, this cross-sectional study was conducted on this age group to assess the following:

- Iodine nutritional status among female adolescents in rural and urban areas of Shahriar region (Iran).
- Knowledge on iodine element, and IDD related complications among female adolescents in Shahriar region (Iran).

## Materials and Methods

### Participants and procedures 

This cross-sectional study was conducted among adolescent school girls aged 14-19 years.

**Figure 1:**
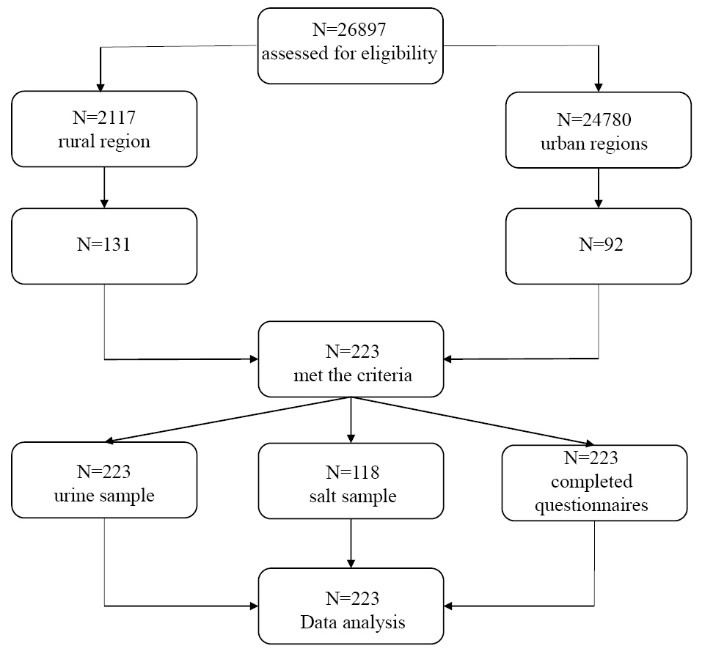
The flowchart of the study.

Students were selected through multi-stage random sampling in Shariar region, Tehran province of Iran, in 2016. There are 318 schools in Shahriar region (254 in urban regions with 24780 students, and 64 schools in rural regions with 2117 students). In this study, 12 schools were randomly selected from both rural and urban regions. Overall, 223 female students were randomly selected from among 26897 students studying in the randomized 12 schools (Figure 1). Apparently healthy female students aged 14-19 years, who lived in rural and urban parts of Shahriar, studying in all types of schools were included in this study. Those who suffered from thyroid or renal diseases, had a history of treatment with thyroid hormones, took supplements containing iodine during the preceding 6 months, or were not willing to participate in the study, were excluded.

Three questionnaires were completed for each student: a demographic questionnaire (to obtain data on demographic characteristics of the subjects), food frequency questionnaire (FFQ) (to collect data on iodine intake), and a questionnaire to assess knowledge about iodine element and IDD. The FFQ consisted of 168 food items, and was validated for estimation of nutrients intake in Tehran Lipid and Glucose study (Tehran, Iran). This FFQ is commonly used in nutritional studies in Iran. Iodine-rich food items were selected for analysis.^23,24^

After being provided with a full explanation of the aim and protocol of the study, the subjects were asked to complete the knowledge questionnaire following the instructions. Knowledge questionnaire consisted of 25 questions (16 questions were about iodine element and its importance in body health, goiter and other IDD complications, iodine-rich food sources, iodine fortification and enrichment, and the knowledge resource of the participants; 9 questions asked about iodized salt consumption). The responses to each question were scored as 0, 0.5 and 1; the range of the total knowledge score was 0-25. The knowledge scores were then categorized based on quartiles.

To determine iodine content of the salts used by the households, the students were asked to bring with them a sample of salt (15 g) in a plastic bag on the sampling day (N = 118). Also, 10 ml of non-fasting urine sample collected in a plastic cup was provided by the students, transferred in an ice box to the laboratory of Endocrinology and Metabolism Research Institute of Shahid Beheshti University of Medical Sciences (Tehran, Iran), and stored at -20°C until analysis.

### Measures

Urinary creatinine and iodine were assessed based on Jaffe’s method^25^ and Sandal-Kalthoff method,^26^ respectively. In the present study, casual urine sample was collected by the students. Urinary iodine secretion fluctuates over 24 hours; therefore, a urinary indicator with fixed concentration over the day is required, so that the urinary iodine excretion can be compared between individuals. For this purpose, daily creatinine excretion was measured for the individuals, as well. Then, the ratio of casual urinary iodine to urinary creatinine excretion was estimated for the students, which allowed for comparisons between the study subjects.^27,28^ This indicator has been used in earlier studies too.^29-34^

MUI values were used for determining iodine status of the students according to the WHO classification method: <20 µg/L as “*Severe deficiency*”, 20-49.9 µg/L as “*Moderate deficiency*”, 50-99.9 µg/L as “*Mild deficiency*”, 100-199.9 µg/L as “*Adequate*”, 200-299.9 µg/L as “*Above requirements”* and ≥300 µg/L* as “Excessive*”.^9^ To measure salt iodine,^35^ iodine was first released by adding H_2_SO_4_ to the salt. To increase iodine solubility in water, KI was added. Finally, the solution was titrated by Na_2_S_2_O_3_. The amount of Na_2_S_2_O_3_ used for titration was used to estimate salt iodine concentration based on the following equations:

IO3- + 5I-+ 6H+→ 3I2 +3H2

Na2 S2O3 + I2 → 2NaI +NA2S4O6

### Statistics

All statistical analyses were performed using SPSS software version 17 (SPSS Inc., Chicago, IL, USA). The distribution normality of continuous variables was tested using Kolmogorov-Smirnov test. One sample t-test was used to compare MUI with the standard values. Comparison of MUI between categorical variables was performed using independent samples t-test and one-way analysis of variance (ANOVA); Kruskal–Wallis H test was used to compare non-normal variables between age groups and regions. To assess the association between urinary iodine levels and knowledge status as well as weekly frequency consumption of goitrogens and iodine-rich foods, chi-square and regression analysis tests were applied, adjusting for the confounders. *P* value less than 0.05 was considered as statistically significant.

## Results

All the study subjects (223 students) completed the questionnaires and provided urinary samples; there were no withdrawals from the study, and data from all the participants were included in the analyses (Figure 1). Table 1 presents the characteristics of the students.

**Figure 2:**
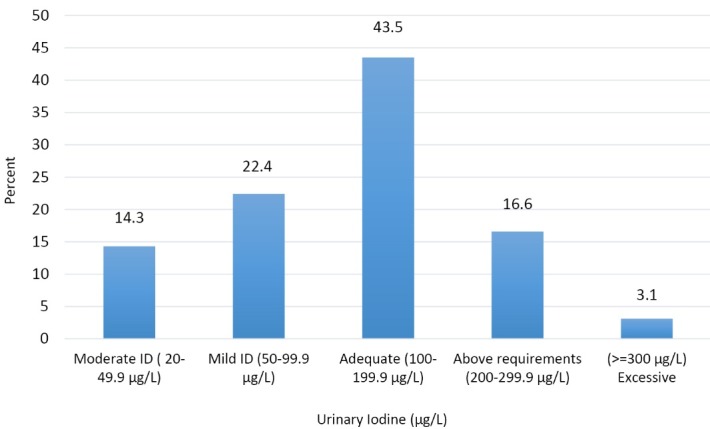
Distribution of urinary iodine concentrations (𝜇g/L) among adolescent school girls based on WHO criteria.

Median and mean (95% CI) of urinary iodine and creatinine concentrations were 129 µg/L, 137.62 µg/L (95% CI: 126.28-148.95) and 1.72 g/L, 1.86 g/L (95% CI: 0.55-3.17) for the study subjects, respectively; the median and mean for the ratio of urinary iodine to creatinine was 70.40 µg/g, 84.12 µg/g (95% CI: 76.21-92.03) (Table 2). According to the WHO criteria, the frequency of mild, moderate, and severe ID was 22.4%, 14.3% and 0% among our subjects, respectively. 43.5% of the students had adequate iodine and 16.6% had urinary iodine level between 200-299.9 µg/L; only 10 subjects (3.1%) had excessive urinary iodine level (≥300 µg/L) (Figure 2). Although MUI level was the highest among 18 year-old girls, no significant differences were found between the age groups. Our results revealed that urinary iodine concentration significantly correlated with urinary creatinine level in each age group, and the degree of correlation coefficient increased with increasing age. There was no statistically significant difference in mean urinary iodine level between urban and rural regions (133.85 µg/L; 95% CI: 117.41-150.29 *vs.* 142.99 µg/L; 95% CI: 128.25-157.37) (Table 2).

Mean salt iodine was 21.69 ppm (95% CI: 19.66-23.72). 20-40 ppm of iodine in salt is considered as “proper concentration”^8^; 63.1% of the salts we studied, contained 20-40 ppm iodine, and 35.9% contained <20 ppm iodine (Table 3). In addition, we found that only 34.1% of the families properly stored the iodized salt; 39.9% of the families added iodized salt to the food at the beginning of the cooking process, while 25.1% of them added the salt at the end of the cooking, and only 18.8% used it at the table. There was no association between salt iodine content and urinary iodine excretion. Urinary iodine excretion was associated with the type of salt used for food preparation and at the table; 78% of the subjects with moderate ID consumed the same salt during food preparation and at the table (*P *= 0.048). The subjects who purchased salt from authorized (reliable) places showed better iodine status (*P *= 0.023). Table 4 presents intake of iodine-rich and goitrogen food sources among subjects with different urinary iodine status. No relationship was found between iodine-rich and goitrogens food sources, and urinary iodine excretion or ratio of urinary iodine to creatinine, even after adjusting for the confounder variables including residency, parents’ job, educational level, and socio-demographic class (Table 5). Results for knowledge on iodine element and IDD revealed that the mean knowledge score was 12.7 ± 3.44, and the proportion of subjects with poor and fair knowledge was 25.1% and 24.2%, respectively. Our results failed to show any association between residency or age group, and knowledge status; however, better knowledge was significantly associated with studying natural sciences at school, compared with other fields (such as mathematics, art, etc) (83.9% *vs.* 16.1%; *P *= 0.012). In addition, knowledge status was better among those who had moderate ID (17.4%), and those with adequate iodine intake (30.4%) (*P *= 0.023). The knowledge sources reported by the participants were radio and TV (39.4%), books and newspaper (11.5%) and healthcare staff (19.4%). A significant association was found between knowledge sources and urinary iodine excretion (*P *= 0.009). Assessment of knowledge on iodine element and its related issues showed a relatively poor knowledge of the students about goiter, iodine element, dietary iodine sources, ID complications, and factors influencing iodine retention (19.3%, 10.3%, 24.2%, 6.3% and 7.2%, respectively).

## Discussion

Since mid-1990s, many countries including Iran, have started national programs to reduce and combat IDD through salt iodization programs and regular assessments of Iodine intake of populations.^36^ ID has been known as a major health problem in Iran, and the national committee for control and prevention of IDD was established in (1989) in this country. After one decade, iodine intake assessment revealed a significant improvement among school aged children in 1995, and Iran was considered as a country free from ID; however, the results of the fourth National Surveillance in 2007 indicated a remarkable decrease in MUI excretion in some regions of Tehran including Shahriar.^37^

In this study, MUI excretion of the students was 129 µg/L, 36.7% of them had mild to moderate ID, and 14.3% had urinary iodine <50 µg/L. In 1995, MUI excretion in Shahriar region was185.13 µg/L, and less than 10% had MUI <50 µg/L.^19^ Several studies carried out during the recent years in Iran, have revealed similar findings. In 1997, Ravanshad et al reported that MUI excretion was 199.5 µg/L among107 students aged 14-18 years in Shiraz.^38^ Another study among 100 students in Fariman, aged 6-18 years, found that MUI excretion was 110 µg/L, and 45.1% of the students had urinary iodine excretion <100 μg/L.^39^ In Urmia, MUI was 143 µg/L among girls aged 10-17 years.^40^ In 2014, MUI and the ration of MUI to urinary creatinine among 12-25 year-old people in Philippine were reported to be 355.3 µg/L and 279 µg/g, respectively.^32^ In England, MUI was 63 µg/L among 57 women aged 19-45 years, and 31.8% of the study subjects had urinary iodine excretion <50 µg/L.^41^ Among 634 girls aged 12-13 years and 246 women aged 18-40 years in North-East Italy, MUI excretion were 77 µg/L and 55 µg/L, respectively.^10^ In the present study, only 3.1% of the female adolescents showed excessive urinary iodine excretion (i.e. equal or greater than 300 µg/L). No studies reported excessive MUI excretion, and therefore we could not compare our results with other studies, for that. In general, it seems that despite salt iodization programs over recent decades, MUI excretion has unfortunately declined in Iran, which may be alarming; this possibly implies that proper IDD surveillances are lacking in Iran, which in turn might result in increased prevalence of IDD, through upcoming years.

We found that 63.1% of the consumed salt had acceptable iodine content. According to the last survey in Tehran (2010), 28.6% of the salts had acceptable iodine content (i.e. 20-40 ppm). Salts with inadequate iodine content (i.e. <20 ppm) reduced from 67% in 2010 compared to 35.93% in our study, 2017.^42^ The iodine content of the consumed salt was found to be acceptable among the households of our study; however, improper packaging, storage and distribution, food processing, as well as washing and cooking processes lead to iodine loss of the salts.^43^ In the present study, proper storage of iodized salt was found in 34.1% of the families compared to 47.9% in 2007^44^ and only 12.6% of the households stored iodized salt in dark containers. These findings indicate iodine loss in the enriched salts, and emphasize the importance of supervision and evaluation of iodized salt preparation and distribution; poor knowledge regarding optimum salt storage conditions needs to be targeted when combating ID.^44^ Indeed, we failed to show an association between salt iodine content and urinary iodine excretion (i.e. urinary iodine excretion was not greater in those with greater iodine intake from salt). However, urinary iodine excretion was associated with using different types of salt for cooking and at the table (i.e. 78% of those with moderate ID used the same salt for cooking and at the table); this highlights the effect of environmental factors on iodine retention in salt.

To the best of our knowledge, this study was the first study in Iran which assessed the consumption of iodine-rich and goitrogen foods, and also their associations with urinary iodine excretion. We failed to find any association between food consumption and urinary iodine levels even after adjusting for rural/urban housing and socio-economic status (Table 5). Other studies have shown a correlation between dairy products consumption and iodine status; this is attributed to the fortification of livestock with some minerals such as iodine in those countries.^12,29,45,46^ In Iran, there is no iodine fortification programs for livestock, and marine foods are considered as the natural food sources of iodine. A study in England in 2014 showed that urinary iodine excretion significantly correlated with consumption of milk and dairy products, fish and egg, while reversely associated with meat and soy beverages.^33^ In Italy, MUI was higher among those with higher consumption of milk.^10^ Results of the students’ knowledge on iodine element, its related complications, and its food sources revealed a significant association between knowledge and urinary iodine excretion. Our study subjects showed poor knowledge regarding dietary iodine sources, ID complications and factors influencing iodine retention (24.2%, 6.3% and 7.2%, respectively), which reflects the necessity for nutrition education programs to reach better results in long term programs aiming IDD.

There were some limitations in our study. The clinical assessment of goiter was not performed by a physician; this was a great but inevitable drawback of our study, due to the funding restrictions we dealt with. This study assessed only female adolescent, and was conducted only in Shahriar region with relatively low sample size, indicating that our results could not be generalized to males and other parts of the country. The FFQ used in our study was generally validated for nutritional research among those living in that area and has been frequently used, however it might be better to prepare and use a specific FFQ for IDD in future studies in our country. Hence, further studies for monitoring female adolescents and women in child-bearing age in different parts of the country are recommended. The present study had some strengths as well; it assessed not only iodine status among a relatively large sample of adolescent girls, but also the students’ knowledge on iodine and IDD in an endemic area for IDD.

## Conclusion

Our findings revealed that adolescent girls in Shahriar had relatively poor knowledge on iodine element, and around one third of them suffered from ID. Therefore, there is urgent need for a comprehensive investigation of adolescents’ iodine status; moreover, implementation of nutrition education programs as well as proper iodine interventions are highly recommended to overcome IDD in this age group.

## Ethical approval

The study protocol was approved by the Ethics Committee of Tabriz University of Medical Sciences, Tabriz, Iran (IR.TBZMED.REC. 1395/49), and written informed consent was obtained from one parent of every student.

## Competing interests

The authors declare that they have no conflict of interests.

## Authors’ contributions

ZH was involved in the conception and designing the study, data collection, and manuscript writing and editing. ME performed the data analysis and interpretation, and was involved in manuscript writing and editing. SR and MH supervised the development of work. EV contributed to editing and revision of the manuscript. All authors have read and approved the final version of the manuscript and agree with the order of presentation of the authors.

## Acknowledgements

Authors kindly thank the staff of Education and Training Office in Shahriar (Iran), and the students for their sincere cooperation in data collection. We also thank Research Institute for Endocrine Sciences, Shahid Beheshti University of Medical Sciences (Tehran, Iran) for their cooperation in iodine analysis in Urine and salt.

**Table 1:** Demographic characteristics of the female students

	**Number**	**Percent**
Birth order		
1	102	48.57
2-3	82	39.04
≥4	26	12.39
Family size (No.)		
≤4	102	47.44
5-6	88	40.94
≥7	25	11.62
Age (y)		
14	64	28.7
16	41	18.4
16	43	19.3
17	42	18.8
18	33	14.8
Total	233	100

**Table 2:** Median and Mean (95% CI) of iodine-related parameters by region and age‏ group

	**Urinary Creatinine** **(g/L)**	**Urinary Iodine** **(µg /L)**	**Urinary Iodine/Creatinine** **(µg /g)**	**Salt Iodine** **(ppm)**
Total	1.72^c^ 1.86 (0.55,3.17)^d^	129 137.62 (126.28,148.95)	70.40 84.12 (76.21,92.03)	24 21.69 (19.66,23.72)
Urban	1.62 1.92 (1.75,2.09)	120 133.85 (117.41,150.29)	62.12 81.06 (69.48,92.64)	24.30 22.73 (20.44,25.02)
Rural	1.82 1.79 (1.87,5.45)	136 142.99 (128.25,157.37)	81.74 88.47 (78.89,98.32)	22.20 20.23 (16.57,23.89)
*P* ^a^	0.333	0.438	0.368	0.262
Age (y)	‏	‏	‏	‏
14	1.72 1.75 (0.05,3.55)	124.50 134.06 (115.88,152.24)	63.89 92 (70.6,113.4‏(	22.20 20.61 (17.6,23.62)
15	1.42 1.84 (1.47,2.21)	134 133.6118 (113.71,153.61)	79.62 85.28 (71.08,99.45)	21.20 20.89 (15.62,26.16)
16	1.82 1.78 (1.51,2.05)	118 129.35 (108.21,150.49)	77.47 81.59 (69.42,93.76)	29.60 25.36 (18.72,32)
17	1.62 1.88 (1.59,2.17)	128 150.24 (114.08,186.4)	71.38 85.78 (70.28,101.28)	27 24.42 (19.6,29.24)
18	2.02 2.21 (1.84,2.58)	144.18 144.18 (109.55,178.81)	58.21 68.55 (54.64,82.46)	21.20 21.07 (15.9,26.24)
*P* ^b^	0.294	0.798	0.496	0.608

^a^ Independent samples *t* test, ^b^ANOVA, ^c^Median, ^d^Mean (95% CI).

**Table 3:** Association between urinary iodine level and salt iodine status

**Urinary iodine status**	**Salt iodine (ppm)**		
	**40 >** **(%)**	**20-40** **(%)**	**<20** **(%)**
Adequate (≥100 µg/L)	0	10.8	21.6
Mild deficiency (50-99 µg/L)	0	23.1	21.6
Moderate & Severe deficiency (≤ 49 µg/L)	100	66.1	56.8
Total	0.97	63.1	35.93
P*		0.308	

*Chi-square.

**Table 4:** Iodine status and iodine-rich and Goitrogen food sources

**Urinary iodine status**	**Goitrogens** **n=149**	**Dairy products** **n=124**	**Fish and tuna fish** **n=141**	**Soya bean** **n=168**	**Egg** **n=199**	**Pizza** **n=166**
Moderate & Severe deficiency (≤49 µg/L)	1.77 (0.76-3.88)	33.18 (27.49-39.93)	0. 83 (0.38-1.25)	1 (0.81-2.75)	2 (1-3)	0.25 (0.07-0.56)
Mild deficiency (50-99 µg/L)	1.81(0.86-3)	31.19 (15.54-41.14)	0.52 (0.27-1.08)	1 (0.25-2)	3 (1-6.5)	0.25 (0.05-5)
Adequate (100-199 mg/L)	3.8(1.59-5.72)	37.53 (24-59.54)	0.75 (0.33-1.25)	1 (0.5-2)	2 (1-3)	0.25 (0.05-0.87)
Excess (200-299 mg/L)	2.13 (1.0-6.67)	37.79 (35.21-40.38)	1.02 (0.75-2)	1 (0.25-2)	2 (1-3)	0.25 (0.08-0.75)

**Table 5:** Correlation between Urinary Iodine and Iodine to creatinine ratio with weekly frequency consumption of goitrogenic foods

**Foods**	**Unadjusted**		**Adjusted**	
	**Urinary iodine (µg/L)**	**Urinary iodine /creatinine (µg/g)**	**Urinary iodine (µg/L)**	**Urinary iodine /creatinine (µg/g)**
Goitrogens	-0.06 (0.87)	-0.03 (0.92)	0.32 (0.67)	0.66 (0.19)
Dairy products	0.30 (0.45)	0.44 (0.25)	-1.38 (0.64)‏	-3.24 (0.15)‏
Fish & tuna fish	0.33 (0.51)	0.16 (0.73)	-1.39 (0.56)‏	-2.86 (0.13)
Soy bean	-0.67 (0.20)	- 0.64 (0.19)	-1.33 (0.41)‏	-2.20 (0.10)‏
Egg	-0.34 (0.42)	-0.03 (0.92)	-0.11 (0.98)‏	0.01 (0.94)‏
Pizza	-0.41 (0.44)	-0.57 (0.27)	3.24 (0.58)‏	6.42 (0.15)

Adjusted for residency and parent’s job and educational level.
